# The Growth and Scope of Voluntary Hospitals in London

**Published:** 1897-09-11

**Authors:** 


### The Institutional Workshop.

### THE GROWTH AND SCOPE OF VOLUNTARY HOSPITALS IN LONDON.

From a Correspondent.

### VIII.—HOSPITAL INTERIORS IN THE EIGHTEENTH CENTURY.

Descriptions of former hospital work are not numerous. A visit to the wards was not the pleasant experience of modern times; they were never entered by those above the class of the patients and their friends, rarely by the governors charged with that duty, and we all know that the hospital officials themselves have never been prodigal of information respecting their institutions. The report of John Howard, who went over every part of every building dedicated to the sick in the course of his inspection in 1788, is the best account we have of the London hospitals in early days. He found much to blame, it is true, but the leading defects, want of air and cleanliness, though conspicuously unfortunate in their effects on recovery, were common at that time to the abodes of rich and poor alike, and it has never been found that any institution can maintain for long together a higher standard in such matters than prevails in the more enlightened part of the community at large. How was it possible, for example, that the free admission of fresh air night and day, the scrubbing of floors, and the regular administration of baths could be enforced when medical officers, lay officials, and patients were alike convinced of the unimportance or actual harmfulness of such details? A few of Howard's criticisms may be noted, throwing much light on hospital usages of the time.

London Hospital.—Wards 20 ft. wide and 12 ft. high, containing 18 beds, which have no testers; square apertures over the door; no cisterns for water; the vaults often offensive; medical and chirurgical patients are together. In a dirty room in the cellar there is a cold and a hot bath, which seem to be seldom used.

St. Bartholomew's.—The wards being double have not the advantage of opposite windows; clean and not offensive except the men's four foul wards, which are on the uppermost storey, and had not one window open. (This was September 19th.) The wards lofty, 22 ft. wide, and in each about 15 beds. Bedsteads wood and testers, though lofty, a harbour for dust and lumber. Beds not crowded, and wards quiet. A large cold bath, and room adjoining for warm bath. To each ward there is a sister and a nurse; the former has a room adjoining, but no window into her ward.

Middlesex Hospital in Marybone.—Sixteen wards, of which four only are occupied, funds being low. Rooms close and dirty, except one called the founder's room. Bedsteads and wooden testers old; the house wants whitewashing, and has an air of poverty.*

St. Thomas's.—Some of the wards only 18 ft. wide. Bedsteads iron, and very properly detached from wall; no testers, but semi-circular irons for curtains in winter; wards fresh and clean, except the three foul wards, in which were 53 men and 27 women; these were very offensive, and had not a window open. There were no water-closets.

* This report much distressed the authorities at Middlesex, and set hem scrubbing and whitewashing with praiseworthy energy.

Guy's Hospital, in Southwark.—Wards in general too low, the height of some only 9½ ft. In several of the wards (each containing about 30 beds) the bed and testers are wood, and infested with bugs. In the new wards, which were clean and fresh, are iron bedsteads and hair beds; the window at the upper end opens from the ceiling to the floor, and there are also ventilators in the ceiling which communicate with the chimneys of the wards over them. The water-closets in the new wards are of the best construction, and not in the least offensive. Here are excellent baths in neat and clean rooms. Improvements in progress.

Westminster Hospital, in James Street.—Wards in general only 17 ft. wide; beds parallel and close to the walls, with wooden testers; floors sanded; walls dirty; most of the upper sashes not moveable. A sum paid every year for the destruction of bugs.

St. George's.—Wards 22½ ft. wide, and 10½ ft. high. Too close, especially the men's, which were very offensive, all windows being shut (September 22). Windows too small and too distant from ceiling; bedsteads old, have testers; beds stand close to the wall; floors sanded; walls wanted whitewashing; a good cold bath, not used.

British Lying-in, Brownlow Street.—Six wards, and in each six beds. Wards clean and quiet; kitchen and pantry clean; house old and wants whitewashing; ceilings too low.

City of London Lying-in.—Eight wards, 17 ft. wide. Wards and beds clean, and over the door of each ward a circular aperture of about 1 ft. in diameter. House wants whitewashing.

The majority of these defects, it will be observed, were structural. The hospitals were not in this respect at all behind their times, and there is reason to believe not only that they readily took advantage of new sanitary improvements, but that in some cases they acted as pioneers in initiating reforms.

Howard also notes defects in discipline. Great quantities of beer were allowed to be brought in for the patients under pretended orders from the faculty; too many promiscuous visitors were allowed; and in some hospitals patients seem to have wandered in and out, and even to have resorted to beershops at their own free will. The art of disciplining numbers without severity or apparent effort, which is such a conspicuous feature of our own times, did not then exist, but the degree of order subsisting in hospital wards is often commented on with admiration as contrasted with the condition of other public institutions.

A graver defect was the imposition of heavy fees and securities on patients, whereby many of those most in need were excluded. The nurses' fees in particular, we learn, opened a door to many impositions. These fees and securities were peculiar to the endowed charities.

It seems to have been a common practice early in the century to place more than one patient in a bed. At Middlesex, in 1747, only 15 beds seem to have been available for an average in the wards of 21. But the universal custom of the times in private houses must be the excuse for inflicting this discomfort on the patient, and the accommodation was palatial when contrasted with the Hôtel Dieu, in Paris, where 20,000

patients were admitted yearly, of whom one-fifth perished, and where the patients lay eight in a bed, in different stages of disease, four at one end and four at the other.

The annual death-rate of in-patients at St. Bartholomew's and St. Thomas's was reckoned at about 7 per cent., as against 5 per cent. in the Northampton and Manchester Infirmaries.

We have very interesting details of the dieting in these early times. The poor suffered in their own homes to an extent not in the least realised by the present generation, from the high price, scarcity, and badness of provisions, and were subject to many diseases entirely due to their being habitually badly nourished on improper food. It is sufficient to glance at any of the pictures of low life at this period to perceive the gaunt figures, hollow eyes, and projecting bones of the unhappy rabble portrayed. In especial the faces of the children are unspeakably sad; old before their time, and with that painful look of hunger never absent from the sharp features. Rare indeed are such faces to-day even in the poorest quarters. Food was not only expensive, it was simply not to be had at some seasons; the very taste of it was poisoned to the merciful by the reflection that what they consumed left less for others, and in many charitable households abstinence from bread or meat was systematically practised on certain days in the week with the design of lowering prices. The usual scarcity was intensified after a bad harvest or in any severe season, and such famine times appear from the periodical literature accessible to have recurred with unfailing regularity every two or three years. At such times big funds were hastily raised and injudiciously expended. The emaciated people were gorged on sheep and oxen roasted whole in open spaces, and with gallons of ale; the slums poured out an indescribable rabble of the lowest order of the populace, and for a few days all was lavishness and riot. The respectable poor hid in silence from such scenes, and died in their own homes from cold and hunger.

The semi-famishing condition of the people must be borne in mind in judging the diet offered in the hospitals. It offered a far higher standard relatively to the common food of the people than the diet of modern hospitals; that is to say, the in-patient to-day is, with few exceptions, less profusely fed in the hospital, in proportion to his ordinary diet at home, than the patient of the eighteenth century was, the balance then being altogether on the side of the hospital. True, the hospital diet has seen many improvements, but the people's diet has been completely revolutionised. The following were the ordinary full diets:—

ST. THOMAS'S.—Breakfast: 4 days milk porridge; 3 days water gruel. Dinner: 5 days ½ lb. meat; 2 days 4 oz. butter or 6 oz. cheese. Supper: 1 pt. broth. Beer: 1 qt. winter; 3 pts. summer. Bread: 14 oz.

LONDON.†—Breakfast: 1 pt. milk pottage or water gruel. Dinner: 8 oz. meat. Supper: 6 days broth. Beer: Winter 1 qt.; summer 3 pts. Bread: 12 oz.

ST. BARTHOLOMEW'S.—Breakfast: 1 pt. milk pottage; Thursday, 1¼ pts. Dinner: 8 oz. meat; Thursday, 2 oz. butter and 4 oz. cheese. Supper: Broth. Beer: 3 pints daily. Bread: 12 oz.

MIDDLESEX.—Breakfast: 1 pt. milk porridge. Dinner: 6 oz. cooked meat. Supper: Broth 1 pt. Beer: Winter 1 pt.; summer 1 qt. Bread: 14 oz.

The quantity of malt liquor in some of these dietaries may seem amazing, but it was probably that second infusion from the malt known as "small beer," and contemptuously alluded to by Shakespeare and others as next door to a temperance drink.

It goes without saying that the food was often of indifferent quality and badly prepared; but continual efforts were made to rectify such abuses, and through these hard times the hospitals certainly set an example of liberality to all those who had the care of the poor.

It must be remembered that the century closed in a season of almost unexampled depression in hospital finance, owing to the popularity of the new dispensaries, whose growth we have rapidly sketched. The giving public, prone at all times to follow a new trail with blind enthusiasm, became for a while oblivious to the claims of the older charities. The courage and tenacious perseverance of those who carried on and extended hospital work under these trying conditions is one of the finest features in their history. Point by point public confidence was restored, improvements were effected, new supporters were drawn in to replace defection, until in 1809 the reaction had fairly set in, and Dr. Lettsom could declare out of his full experience that such charities in this Metropolis were more liberally supported than ever was heretofore known.

† Howard stated that at the London Hospital the Jewish patients were truly starved on an allowance of 2 d. a day.

